# PET Imaging of Liposomal Glucocorticoids using ^89^Zr-oxine: Theranostic Applications in Inflammatory Arthritis

**DOI:** 10.7150/thno.40403

**Published:** 2020-02-26

**Authors:** Peter J. Gawne, Fiona Clarke, Keren Turjeman, Andrew P. Cope, Nicholas J. Long, Yechezkel Barenholz, Samantha Y. A. Terry, Rafael T. M. de Rosales

**Affiliations:** 1 School of Imaging Sciences & Biomedical Engineering, King's College London, St. Thomas' Hospital, London, SE1 7EH, UK.; 2 Centre for Inflammation Biology and Cancer Immunology, King's College London, New Hunt's House, London, SE1 1UL, UK.; 3 Laboratory of Membrane and Liposome Research, Department of Biochemistry,Institute for Medical Research Israel-Canada, Hebrew University-Hadassah Medical School, Jerusalem, Israel.; 4 Department of Chemistry, MSRH, Imperial College London, White City Campus, W12 0BZ, London, UK.; 5 London Centre for Nanotechnology, King's College London, Strand Campus, London, WC2R 2LS, United Kingdom, UK.

**Keywords:** Positron Emission Tomography, Nanomedicine, *In vivo* Liposome Imaging, Personalised Nanomedicine, Rheumatoid Arthritis

## Abstract

The encapsulation of Glucocorticoids (GCs) into long-circulating liposomes (LCLs) is a proven strategy to reduce the side effects of glucocorticoids and improve the treatment of inflammatory diseases, such as rheumatoid arthritis (RA). With the aim of supporting the development of GC-loaded LCLs, and potentially predict patient response to therapy clinically, we evaluated a direct PET imaging radiolabelling approach for preformed GC-LCLs in an animal model of human inflammatory arthritis.

**Methods:** A preformed PEGylated liposomal methylprednisolone hemisuccinate (NSSL-MPS) nanomedicine was radiolabelled using [^89^Zr]Zr(oxinate)_4_ (^89^Zr-oxine), characterised and tracked *in vivo* using PET imaging in a K/BxN serum-transfer arthritis (STA) mouse model of inflammatory arthritis and non-inflamed controls. Histology and joint size measurements were used to confirm inflammation. The biodistribution of ^89^Zr-NSSL-MPS was compared to that of free ^89^Zr in the same model. A therapeutic study using NSSL-MPS using the same time points as the PET/CT imaging was carried out.

**Results:** The radiolabelling efficiency of NSSL-MPS with [^89^Zr]Zr(oxinate)_4_ was 69 ± 8 %. PET/CT imaging of ^89^Zr-NSSL-MPS showed high uptake (3.6 ± 1.5 % ID; 17.4 ± 9.3 % ID/mL) at inflamed joints, with low activity present in non-inflamed joints (0.5 ± 0.1 % ID; 2.7 ± 1.1 % ID/mL). Importantly, a clear correlation between joint swelling and high ^89^Zr-NSSL-MPS uptake was observed, which was not observed with free ^89^Zr. STA mice receiving a therapeutic dose of NSSL-MPS showed a reduction in inflammation at the time points used for the PET/CT imaging compared with the control group.

**Conclusions:** PET imaging was used for the first time to track a liposomal glucocorticoid, showing high uptake at visible and occult inflamed sites and a good correlation with the degree of inflammation. A subsequent therapeutic response matching imaging time points in the same model demonstrated the potential of this radiolabeling method as a theranostic tool for the prediction of therapeutic response - with NSSL-MPS and similar nanomedicines - in the treatment of inflammatory diseases

## Introduction

Rheumatoid arthritis (RA) is a chronic, auto-immune disease that results in joint inflammation as well as bone and cartilage damage. RA affects approximately 400,000 people in the UK alone 1, with global incidence rates of approximately 1% (75 million people worldwide) 2. It is a highly debilitating disease; causing pain, stiffness, swelling and limited motion to patients, and may lead to disability. RA affects any joint and develops at any age, although is most prevalent in women, being 3 times more likely to suffer from it than men.

The treatment of RA and other inflammatory diseases often includes the use of glucocorticoids (GCs), which are well-established drugs with strong anti-inflammatory properties. GCs, however, are also notorious for their toxicity and wide range of short- and long-term side-effects 3. These include, in the long term: cataracts, high blood sugar/diabetes, infections, osteoporosis and bone fractures; and in the short term: glaucoma, hypertension and psychological effects such as psychosis.

A strategy with proven efficacy in reducing the toxicity of GCs is encapsulation into long-circulating liposomes (LCLs, also known as stealth liposomes). LCLs can passively accumulate in inflamed tissues by exploiting the leakiness of the vasculature as well as inflammatory cell sequestration present in these tissues (ELVIS phenomenon: extravasation through leaky vasculature and subsequent inflammatory cell-mediated sequestration) 4-6. This may lead to preferential accumulation of LCLs at inflamed sites, reducing systemic toxicity and improving the treatment of inflammatory diseases such as RA 4. In order to understand the *in vivo* behaviour of LCLs, including the level of accumulation at inflamed sites, it is important to develop non-invasive imaging techniques for visualising, quantifying, and monitoring their biodistribution over time. This will support the effective selection of the best LCLs for clinical translation 7. In addition, the field of nanomedicine is increasingly becoming aware that the ELVIS and enhanced permeation and retention (EPR) phenomena can be highly variable in humans. For example, the uptake of anti-cancer LCLs in solid tumours (that rely on the EPR phenomenon) is highly heterogeneous in patients, differing not only from person-to-person but also from lesion-to-lesion, even within the same patient 8-10. To overcome this issue, the concept of 'personalised nanomedicine' - in which medical imaging is used in a theranostic approach to predict the efficacy of the nanomedicinal treatment - has been proposed 11. In the context of LCLs, this would involve whole-body *in vivo* tracking of a sub-therapeutic dosage of the liposomal nanomedicine. The treatment of a patient can then be tailored based on the whole-body biodistribution and levels of accumulation of the nanomedicine in inflamed tissues 11. It is expected that implementation of this approach will allow selection of the best nanomedicine candidates for clinical translation, hence optimizing this expensive process. In addition, it should improve their therapeutic clinical efficacy, by providing a method to pre-select only those patients that will truly benefit from the nanomedicinal treatment.

To support the preclinical and clinical development of LCLs as well as personalised nanomedicine approaches, we aim to develop simple radiolabelling tools to track LCLs *in vivo* using positron emission tomography (PET) 7,12-14. This imaging modality is the most sensitive and quantifiable technique available for whole-body nanomedicine tracking over time, in both animals and humans. With this goal in mind, we recently developed a method that allows radiolabelling of liposomes without modification 12-14. By utilising radio-ionophore complexes such as [^89^Zr]Zr(oxinate)_4_ (^89^Zr-oxine) to transport radionuclides across the lipid bilayer, we found that chelating groups on the incorporated drugs allowed efficient trapping of the radionuclides inside the liposomal core for *in vivo* PET imaging. This mechanism allowed us to radiolabel and track preformulated PEGylated liposomal alendronate (PLA) and DOXIL^®^ *in vivo* 12,13.

**Figure 1:**
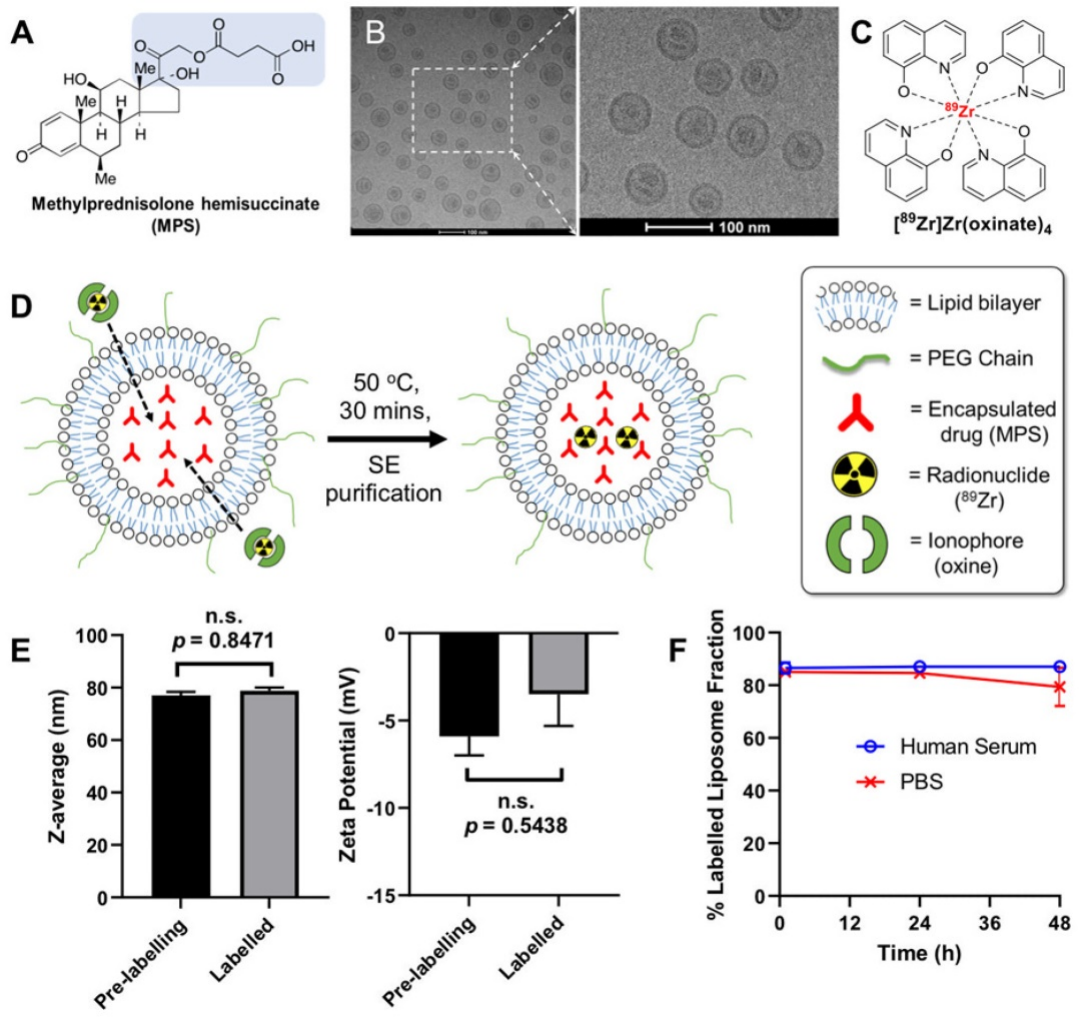
(A) Chemical structure of the glucocorticoid used in this study, methylprednisolone hemisuccinate (MPS). The predicted chelating groups on the drug are highlighted in blue. (B) TEM images of the MPS-LCL, NSSL-MPS. (C) Chemical structure of the radio-ionophore complex [^89^Zr]Zr(oxinate)_4._ (D) Scheme showing the radiolabelling of liposomes using [^89^Zr]Zr(oxinate)_4_. (E) Size measurements of NSSL-MPS (left, n.s. = not significant; P = 0.8471) and zeta potential measurements (right, n.s.; P = 0.5438) before and after radiolabelling with [^89^Zr]Zr(oxinate)_4._ Data represents n = 1, in triplicate. (F) Stability of ^89^Zr-NSSL-MPS in PBS and serum stability (37 ºC) across 48 h (n = 3). All error bars represent mean ± SD.

In this study we extended this methodology for the radiolabelling and *in vivo* tracking of a PEGylated liposomal glucocorticoid (NSSL-MPS). This LCL encapsulates the well-established GC methylprednisolone hemisuccinate (MPS, Figure 1A) and has shown increased efficacy versus non-liposomal MPS in several animal models of inflammation 5 - particularly in RA 15,16. In this work, NSSL-MPS (Figure 1B) was directly labelled using [^89^Zr]Zr(oxinate)_4_ (structure in Figure 1C) and tracked *in vivo* in a RA mouse model to assess the extent of uptake in inflamed arthritic joints, as well as healthy joints, and investigate whether this imaging method could be used in a theranostic approach.

### Materials and methods

All chemical reagents were purchased from commercial sources. Water (18.2 MΩ·cm) was obtained from an ELGA Purelab Option-Qsystem. IR analysis was carried out using a PerkinElmer Spectrum 100 FT-IR spectrometer. No-carrier-added ^89^Zr (produced at the BV Cyclotron, VU Amsterdam, NL) was purchased from PerkinElmer as [^89^Zr]Zr(oxalate)_4_ in 1 M oxalic acid. Radioactivity in samples was measured using a CRC-25R dose calibrator (Capintec). iTLC-SG and SA chromatography plates were purchased from Agilent, UK and scanned using a PerkinElmer Cyclone Plus Storage Phosphor Imager. PET/CT imaging was performed on a nanoScan *in vivo* PET/CT preclinical imager (Mediso Medical Imaging Systems, Budapest, Hungary). Gamma counting was performed using a Wallac 1282 CompuGamma γ counter. DOXEBO (LC500) was supplied by the Barenholz lab. The human biological samples were sourced ethically and their research use was in accord with the terms of the informed consents under an Institutional Review Board/Ethics Committee (IRB/EC) approved protocol.

### Preparation of PEGylated liposomal methylprednisolone (NSSL-MPS)

PEGylated liposomal methylprednisolone (NSSL-MPS) was prepared as previously described 15,17. Briefly, lipids (hydrogenated soy-bean phosphatidylcholine/cholesterol/PEG-DSPE-2000 of 56.6:38.1:5 mole %; 3:1:1, w/w) were dissolved in ethanol and then were hydrated with 250 mM calcium acetate at 65°C, above the phase transition temperature. The large multilamellar vesicles formed upon lipid hydration were downsised by sequential extrusion at 65°C through polycarbonate filters of decreasing defined pore size, starting with a 400 nm pore size filter and ending with a 50 nm pore size filter, under increasing nitrogen pressure (up to 200 pounds/square inch). Calcium acetate in the external liposome medium was replaced by 10% sucrose (w/v) by a diafiltration step at 4ºC using a Labscale TFF System having a Pellicon XL, 500 K polyethersulfone membrane (Millipore Corp., Billerica, Maine, USA). The nano-liposomes exhibiting transmembrane calcium acetate gradient were remotely-loaded with MPS sodium salt as previously described 17.

### NSSL-MPS radiolabelling with [^89^Zr]Zr(oxinate)_4_

[^89^Zr]Zr(oxinate)_4_ was synthesised as previously reported 18,19; full details are provided in the supplementary information. The radiolabelling of NSSL-MPS was based on our previously reported method 12. Briefly, [^89^Zr]Zr(oxinate)_4_ was incubated with NSSL-MPS liposomes for 30 min at 50 ^o^C with regular agitation. The mixture (150 - 200 µL) was left to cool to room temperature and then loaded onto a G-25 Minitrap size exclusion (SE) column (GE Healthcare, pre-washed with 8 mL of saline), followed by the addition of saline (300 - 350 µL), to make the final volume of sample loaded into the column equal to 0.5 mL. The ^89^Zr-labelled liposomes (^89^Zr-NSSL-MPS) were eluted from the column by the addition of saline (750 µL). The eluate and column were γ-counted and the liposome labelling efficiency (LE) was calculated as:

%LE = [liposome fraction CPM / (liposome fraction CPM + column CPM)] × 100.

where CPM = counts per minute

The *in vitro* stability of ^89^Zr-NSSL-MPS was evaluated as indicated below by incubation in human serum and PBS (1:2 v/v liposome/serum, 0.11 mg/mL MPS, 0.91 mg/mL lipids) over 48 h at 37 ^o^C and room temperature, respectively.

**For PBS stability:** At 1 h, 24 h, and 48 h, a 0.25 mL aliquot of the ^89^Zr-NSSL-MPS dispersion in PBS was added to a 10 mM aqueous solution of DTPA (50 µL) and left to sit for 5 min. The resulting solution was then loaded onto a G-25 Minitrap size exclusion column (GE Healthcare, pre-washed with 8 mL of saline), followed by the addition of aliquots of saline (200 µL). The ^89^Zr-labelled liposomes (^89^Zr-NSSL-MPS) were eluted from the column by the addition of saline (800 µL). The eluate and column were γ-counted and the stability was calculated as:

% Labelled liposome fraction = [liposome fraction CPM / (liposome fraction CPM + column CPM)] × 100.

where CPM = counts per minute

**For human serum stability:** At 1 h, 24 h, and 48 h, 0.2 mL of the resulting mixture was injected into a size exclusion chromatography column (Superose 6 Increase 10/300 GL column; GE Healthcare, UK) at a flow rate of 0.5 mL PBS buffer/min. This method has been previously used to efficiently separate liposomes from serum proteins and small molecules 12,20,21. Using our system, liposomes elute as a single peak between fractions 8 and 11 mL, and serum proteins elute between 18 and 25 mL. All fractions were γ-counted, and the column was monitored to confirm the absence of remaining activity. To measure the stability of ^89^Zr-NSSL-MPS, the following formula was used:

% Labelled liposome fraction = [liposome fractions CPM / (liposome fractions CPM + serum protein fractions CPM)] × 100

where CPM = counts per minute

### Arthritis induction and animal monitoring

**Figure 2:**
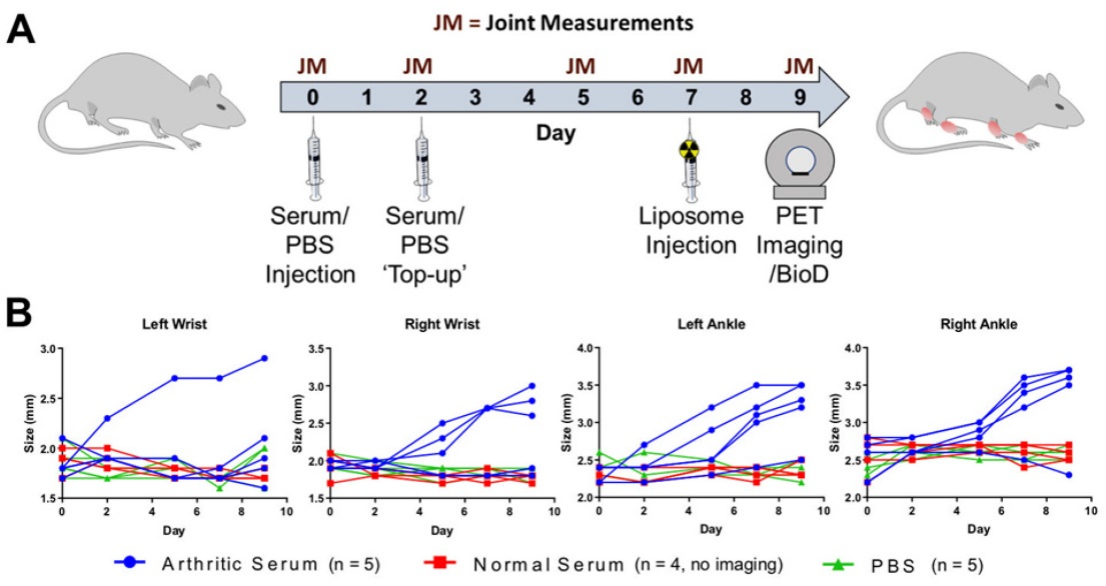
(A) Experimental schedule used for the induction of inflammatory arthritis in the K/BxN STA mouse model and subsequent PET/CT imaging study; (B) Plot of the caliper measurements in each of the wrists and ankles over time.

Inflammatory arthritis was induced using the K/BxN serum transfer arthritis (STA) model 22,23. Briefly, 9-week old female C57Bl/6J mice (n = 5) were injected *i.p*. with arthritogenic serum (150 µL, 50 % v/v PBS, obtained from K/BxN transgenic mice) on day 0, followed by an additional injection on day 2 (Figure 2A). Control groups were injected *i.p* as above with non-arthritogenic serum (150 µL, 50 % v/v PBS; n = 4) or PBS (150 µL; n = 5). The animals were weighed and visual inflammation scores assigned on each day post-serum injection, along with caliper measurements being performed on the wrists and ankles on days 0, 2, 5, 7 & 9 (Figure 2B). Caliper measurements were used to calculate joint swelling as:

Joint size on day X - Joint size on day 0 = Joint Swelling on day X

### PET/CT imaging

Animal imaging studies were ethically reviewed and carried out in accordance with the Animals (Scientific Procedures) Act 1986 (ASPA) UK Home Office regulations governing animal experimentation and the GSK Policy on the Care, Welfare and Treatment of Animals. All mice were anesthetised with isofluorane (2 - 3 %) during all imaging sessions. For injection, the lipid concentration of ^89^Zr-NSSL-MPS was adjusted to 10 mg/mL by the addition of DOXEBO (empty PEGylated liposomes). On day 0 (see Figure 2A for schedule) ^89^Zr-NSSL-MPS (1.3 MBq, 100 μL saline, 2 mg/kg MPS dosage, 1 mg/mouse lipid dose) was injected *i.v.* into the mice (n = 14) at t = 0 h. PET/CT imaging was performed at t = 48 h p.i. on day 9 (n = 4, RA; n = 4, PBS) for 60 min on a nanoScan *in vivo* PET/CT preclinical imager (Mediso Medical Imaging Systems, Budapest, Hungary). All PET/CT data sets were reconstructed using a Monte Carlo based full 3D iterative algorithm (Tera-Tomo, Mediso Medical Imaging Systems, Budapest, Hungary). Decay correction to time of injection was applied. CT images were obtained with 55 kVp tube voltage, 1200 ms exposure time in 360 projections. All the images were analysed using VivoQuant software (inviCRO, USA).

### *Ex vivo* biodistribution

Biodistribution studies were carried out in accordance with UK Home Office regulations governing animal experimentation. Immediately after the PET/CT imaging studies (at t = 49 h p.i. on day 9) mice (n = 14) were culled by cervical dislocation whilst under anaesthesia, and the organs of interest were dissected. Each sample was then weighed and counted with a γ counter, together with standards prepared from a sample of the injected ^89^Zr-NSSL-MPS to obtain percentages of the injected dose per mass values (% ID/g) for each organ/tissue.

### Histology

All joints were fixed in 4 % formalin for one week at 4 °C and were processed by UCL IQPath (London, UK) for histologic analysis. FFPE organ blocks were sliced and stained with hematoxylin & eosin. Immunohistochemistry was performed with a Discovery XT system (Ventana Medical Systems) using the DAB Map detection kit (Ventana #760-124). For pre-treatment, CC1 (Ventana #950-124) was used. Sections were stained for neutrophil elastase (anti-Ly6G), macrophages (anti-F4/80) and blood vessels (anti-CD31).

### NSSL-MPS therapy study

Inflammatory arthritis was induced in female 9-week old C57Bl/6 mice (n = 9) as described above, with visual inflammation scores assigned and mouse weights taken on each day post-serum injection (Figure 7A-B) and caliper measurements being performed on the wrists and ankles on days 0, 2, 5, 7 - 14 (Figure 7C-D). At day 7 post-serum injection, all mice were anesthetised with isofluorane (1.5 - 2%) and either NSSL-MPS (100 µL, 25 mg/kg MPS dosage, 4.7 mg/mouse lipid dose; n = 5) or PBS (100 µL; n = 4) were injected *i.v.* into the mice. Both the visual inflammation scores and caliper measurements were blinded after injection of the liposomes (day 8 - 14). Any joints which did not show signs of inflammatory arthritis were excluded from the swelling plots in Figure 7 (data is shown in the supplementary Figure S1).

## Results

### NSSL-MPS can be radiolabelled with [^89^Zr]Zr(oxinate)_4_

The radiolabelling reaction of NSSL-MPS with [^89^Zr]Zr(oxinate)_4_ is shown schematically in Figure 1D. The metastable, neutral and lipophilic [^89^Zr]Zr(oxinate)_4_ complex passively crosses the lipid bilayer of the liposomes releasing the radionuclide inside the aqueous liposomal core. The free ^89^Zr ion is then trapped *via* binding to chelation groups on MPS (Figure 1A). IR spectroscopy of a MPS and ZrCl_4_ mixture (Figure S2) showed the formation of two bands (1550 cm^-1^ and 1450 cm^-1^) corresponding to a Zr-O bond between Zr^4+^ and a carboxylate group (Zr-OOC), indicating that ^89^Zr binds *via* the carboxylate on the hemisuccinate moiety. After 30 min incubation at 50 ^°^C, and subsequent purification using a SE column, the labelling efficiency (LE) was found to be 69.3 ± 7.7 % (n = 6). No effect on the size (hydrodynamic diameter) of NSSL-MPS or its surface charge (z-potential) was observed (Figure 1E, Table s1 in the supplementary information). The hydrodynamic diameter of NSSL-MPS was 77 ± 1.4 nm and 78.8 ± 1.2 nm before and after labelling respectively (*P* = 0.8471, no significance), and the z-potential was -5.9 ± 1.1 mV and -3.5 ± 1.8 mV before and after the labelling of NSSL-MPS with [^89^Zr]Zr(oxinate)_4_ (*P =* 0.5438, no significance). The *in vitro* radiolabelling stability of ^89^Zr-NSSL-MPS was tested by incubation with PBS at room temperature and with human serum at 37^ o^C over 48 h. The radiolabelled liposomes were then separated from non-liposome associated ^89^Zr in PBS by passing them down a G-25 SE column. ^89^Zr-NSSL-MPS was separated from serum proteins using a SE column on a fast protein liquid chromatography (FPLC) system. Figure 1F summarises the stability of ^89^Zr-NSSL-MPS over 48 h. At 1 h incubation, the amount of ^89^Zr associated with NSSL-MPS was 85.0 ± 1.7 % and 86.5 ± 2.1 % in PBS and serum, respectively. Whilst the stability in serum remained unchanged, with 87.0 ± 0.4 % stability at 48 h, there was a slight decrease for ^89^Zr-NSSL-MPS in PBS with 79.4 ± 7.3 % stability at 48 h.

### ^89^Zr-NSSL-MPS can be tracked *in vivo* using PET imaging, revealing high uptake into both exposed and occult inflamed joints in an animal model of human inflammatory arthritis

After the successful radiolabeling of NSSL-MPS, the ability of these liposomes to accumulate at arthritic joints was assessed using PET imaging. Figure 2A shows the experimental schedule used. Inflammatory arthritis was induced in healthy female C57Bl/6J mice (n = 5) by intraperitoneal injection of K/BxN serum on days 0 and 2, whereas control groups were either injected with non-arthritogenic (normal) mouse serum (n = 4) or PBS (n = 5). The size of the wrists and joints of each animal were monitored to track the progress of the disease. Figure 2B shows the size for each of the wrists and ankles over time for all animals, demonstrating that only joints of the STA mice group showed an increase. This model induces an asymmetric pattern of inflammation, with more inflamed joints in the right hand side of the animal (7/10) than in the left side (5/10), and more inflammation in ankles (8/10), than in wrists (4/10).

**Figure 3:**
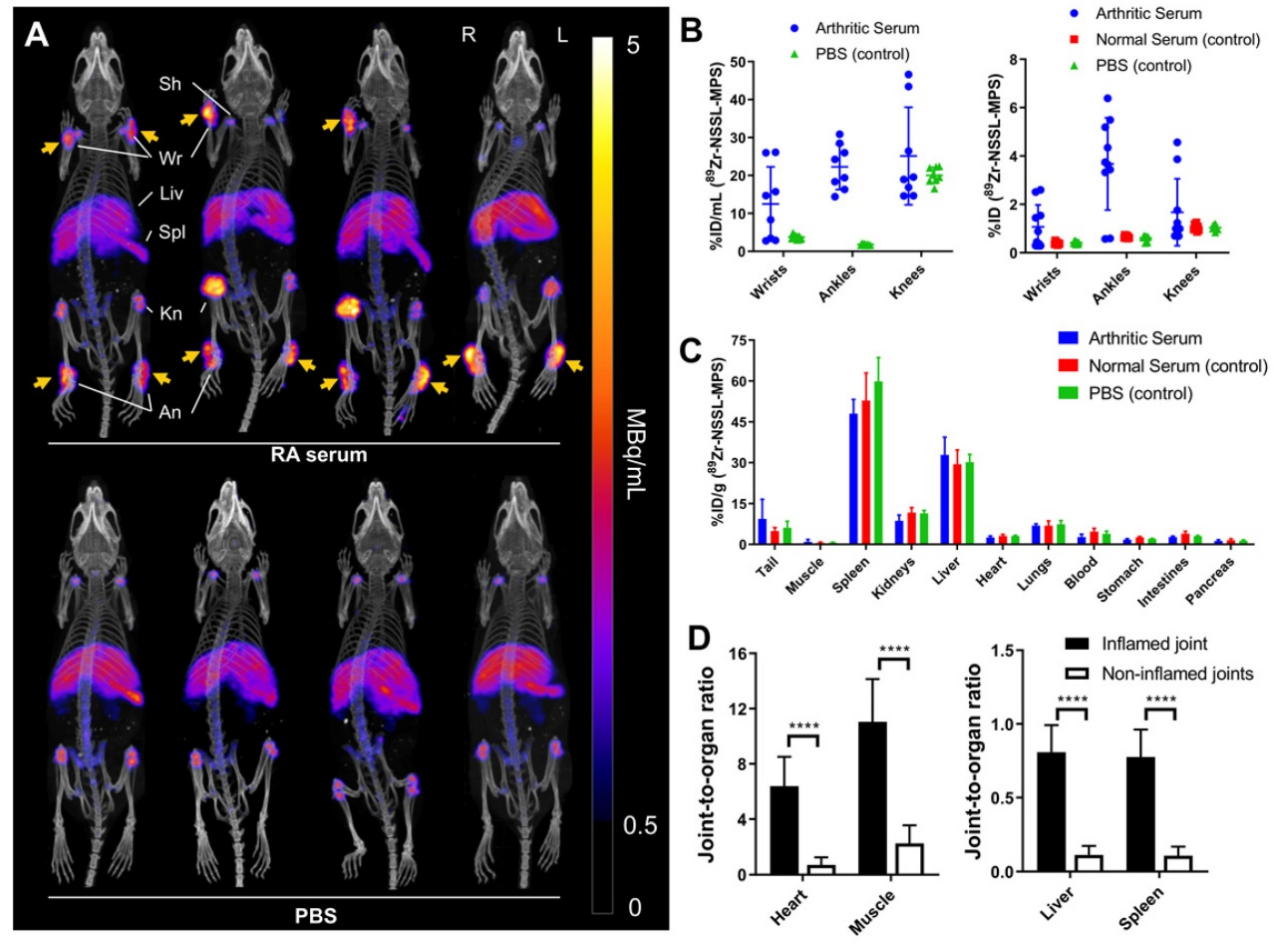
(A) PET/CT maximum intensity projections of C57Bl/6J mice with (top row) and without (bottom row) serum induced inflammatory arthritis. Swelling in the joints of the arthritic mice is clearly demonstrated by liposomal uptake in the wrists and ankles (orange arrows). (B) Uptake of ^89^Zr-NSSL-MPS in wrists, ankles and knees of mice injected with either arthritic serum or PBS based on image quantification (% ID/mL, left graph; % ID/mL = percentage of the total injected dose per mL of tissue) and *ex vivo* biodistribution (% ID, right graph). (C) *Ex vivo* biodistribution at 48 h p.i. of ^89^Zr-NSSL-MPS. (D) Joint-to-organ ratio for heart, muscle (left graph), liver and spleen (right graph) for inflamed and non-inflamed joints. Inflammed joints are defined as those with swelling >0.5 mm. **** = P <0.0001; All error bars represent mean ± SD.

On day 7 post-arthritic serum or controls (PBS/normal serum) injection, ^89^Zr-NSSL-MPS (1.3 MBq, 100 µL saline, 2 mg/kg MPS dosage, 1 mg/mouse lipid) was injected intravenously into all mice. This was followed by PET imaging 48 h later (day 9) of four mice from the STA and PBS groups. This imaging time point was chosen to allow the liposomes to be in circulation during the peak of inflammation, which occurs at days 8 - 9 post induction,^24^ and taking into account that the circulation half-life of NSSL-MPS liposomes is t_1/2_ = 34 h 15. This long circulation half-life is consistent with preliminary PET imaging studies in healthy mice (Figure S4). *Ex vivo* biodistribution was carried out on all mice after the imaging sessions. PET/CT imaging of the STA mice (Figure 3A) showed high ^89^Zr-NSSL-MPS uptake at inflamed joints (3.6 ± 1.5 % ID; 17.4 ± 9.3 % ID/mL, n = 12 combining ankles and wrists, Figure 3B). In contrast, control groups lacking joint inflammation showed low uptake (0.5 ± 0.1 % ID, n = 36; 2.7 ± 1.1 % ID/mL, n = 16). ^89^Zr-NSSL-MPS uptake into non-target organs was similar across all groups, with high uptake in spleen (53.6 ± 8.8 % ID/g, n = 14) and liver (30.9 ± 4.7% ID/g, n = 14) (Figure 3C). Furthermore, plotting the joint-to-organ (heart, muscle, liver and spleen) ratio of inflamed and non-inflamed joints (non-inflamed defined as joints with swelling <0.5 mm on day 9) showed a significantly higher ratio for inflamed joints (Figure 3D).

**Figure 4:**
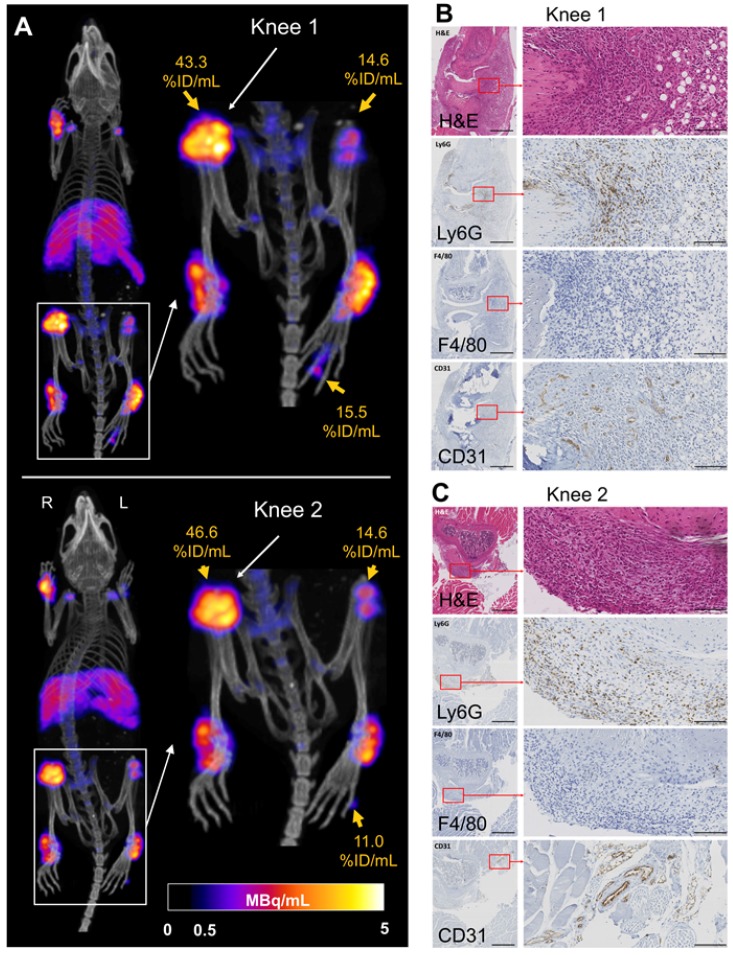
(A) PET/CT MIP of C57Bl/6J mice with suspected inflamed right knees (Knee 1 and Knee 2) and hind paw digit uptake. B) H&E and immunohistochemistry showing increased vascularisation (anti-CD31) and neutrophil infiltration (anti-Ly6G) and staining for macrophages (F4/80) for Knee 1 and (C) Knee 2. For the left panels magnification is 4x with the black bar denoting 500 µm; for the right panels magnification is 30x with the black bar denoting 100 µm.

Interestingly, ^89^Zr-NSSL-MPS PET detected unexpected high levels of uptake in joints that were not detected visually. Particularly, very high accumulation was detected in the right knee of two mice from the disease group (45.0 ± 1.6 % ID/mL; n = 2, Figure 4A), as well as in two hind paw digital joints (13.2 ± 2.4 % ID/mL; n = 2, Figure 4A). These levels of uptake are significantly higher compared to non-inflamed knees (19.4 ± 3.1 % ID/mL; n = 14) and hind paw digital joints (2.5 ± 1.5 %ID/mL; n = 8). Histological confirmation of inflammation was obtained for the knees (Figure 4), showing characteristic high levels of neutrophil infiltration and increased angiogenesis (Figure 4B-C) 24.

### PET imaging and *ex vivo* biodistribution studies demonstrate a correlation between ^89^Zr-NSSL-MPS uptake levels in joints and inflammation

**Figure 5:**
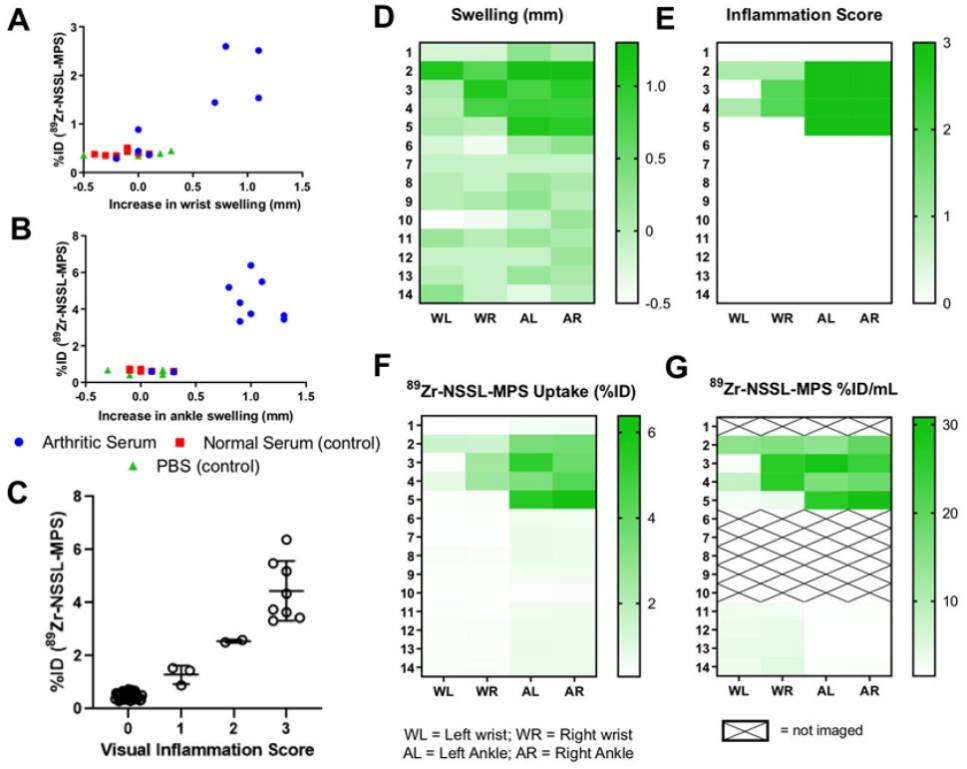
(A) Plot of %ID of ^89^Zr-NSSL-MPS versus swelling of wrists, (B) versus swelling of ankles, and (C) versus visual inflammation scores (all joints from all groups) at day 9 post serum injection; (D-G) Heatmaps showing (D) joint swelling, (E) visual inflammation scores, (F) ^89^Zr-NSSL-MPS uptake (%ID) from ex-vivo biodistribution and (G) image-quantified uptake (%ID/mL) (G) for all mice at day 9.

A clear relationship between increased joint swelling/inflammation and high ^89^Zr-NSSL-MPS uptake was observed (Figure 5A-C). Joint swelling (defined as those >0.5 mm) was only observed in the disease group, which showed high ^89^Zr-NSSL-MPS uptake of 2.0 ± 0.5 % ID (n = 4) in wrists and 4.4 ± 1.0 % ID (n = 8) in ankles compared to non-inflamed joints which had uptake of 0.4 ± 0.1 % ID (n = 24) and 0.6 ± 0.1 % ID (n = 20) for wrists and ankles, respectively. The correlation is also evident when comparing the data from both joint swelling measurements and visual inflammation scores with the *in vivo* uptake of ^89^Zr-NSSL-MPS based on PET imaging quantification, and *ex vivo* biodistribution data (Figure 5D-G, all data from day 9 post serum injection).

### The biodistribution of unchelated^ 89^Zr and its uptake in inflamed joints is significantly different to ^89^Zr-NSSL-MPS

**Figure 6:**
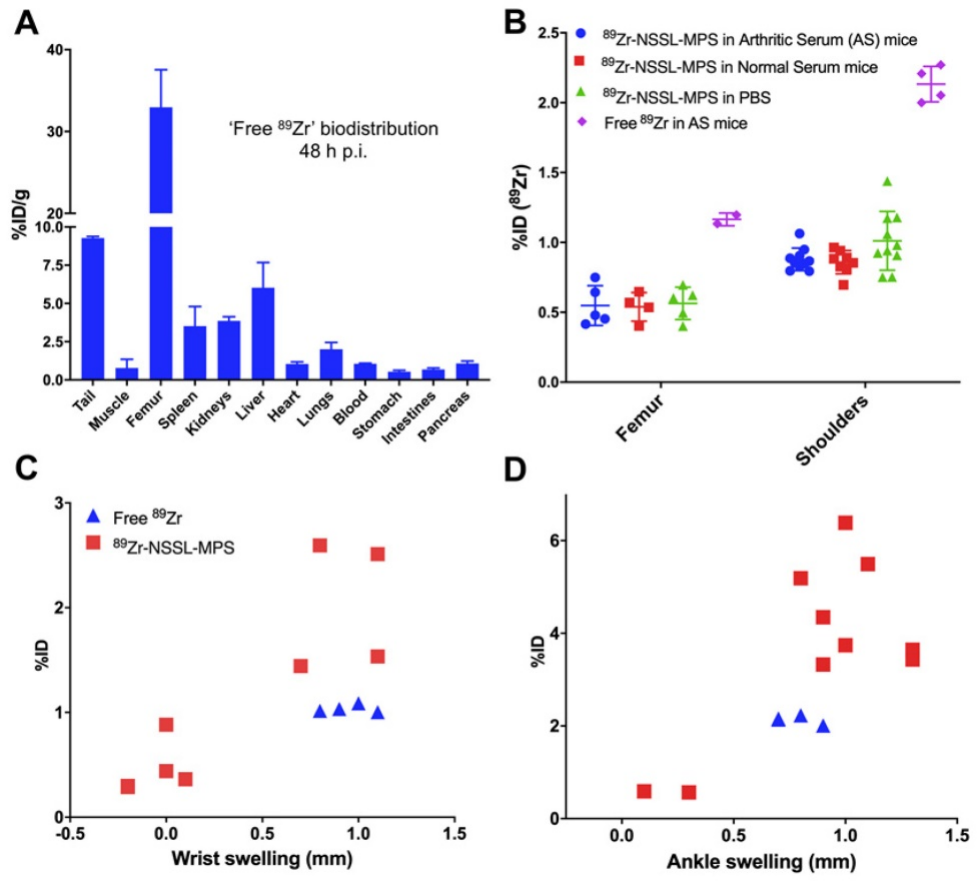
(A) *Ex vivo* biodistribution of unchelated ^89^Zr in STA mice 48 h p.i. (B) Uptake (% ID) of ^89^Zr-NSSL-MPS in the femur and shoulders of RA and control mice (blue dots, red squares and green triangles) and uptake (% ID) of unchelated ^89^Zr NSSL-MPS in the femur and shoulders of STA mice (purple diamonds). (C) Plot of % ID of ^89^Zr-NSSL-MPS and unchelated ^89^Zr versus swelling of wrists and (D) versus swelling of ankles at day 9 post serum injection. All error bars represent mean ± SD.

A concern from the ^89^Zr-NSSL-MPS imaging study (*vide supra*) was the contribution of any potential unchelated ^89^Zr in the uptake into inflamed tissues. To evaluate this we injected mice (n = 2) with STA on day 7 post serum injection with neutralised [^89^Zr]ZrCl_4_ (see supplementary information for experimental details), performing *ex vivo* biodistribution 48 h p.i. (day 9). The biodistribution of [^89^Zr]ZrCl_4_ demonstrated a distinct difference in accumulation from ^89^Zr-NSSL-MPS, characterised by high bone uptake (33.0 ± 4.5 % ID/g) and low uptake (<10 % ID/g) in all other organs - including the liver and spleen (Figure 6A). Additionally, the % ID of [^89^Zr]ZrCl_4_ in the shoulders and femur of STA mice was over double compared with ^89^Zr-NSSL-MPS (Figure 6B) - with the same % ID found for the labelled liposomes in the control serum and PBS groups. A further distinction between the 'free ^89^Zr' and the labelled liposomes could be made when plotting %ID in the joints *vs.* joints swelling (Figure 6C). Whereas liposomal uptake correlates with joint swelling (*vide supra*), the uptake of [^89^Zr]ZrCl_4_ was consistent over increasing joint swelling. Furthermore, the % ID in inflamed joints was consistently lower for [^89^Zr]ZrCl_4_ compared to ^89^Zr-NSSL-MPS; with 1.0 ± 0.0 % ID and 2.0 ± 0.5 % ID respectively for wrists, and 2.1 ± 0.1 % ID and 4.4 ± 1.0 % ID respectively for ankles.

### Treatment with NSSL-MPS shows anti-inflammatory effects in the K/BxN serum-transfer arthritis (STA) mouse model of human inflammatory arthritis

**Figure 7:**
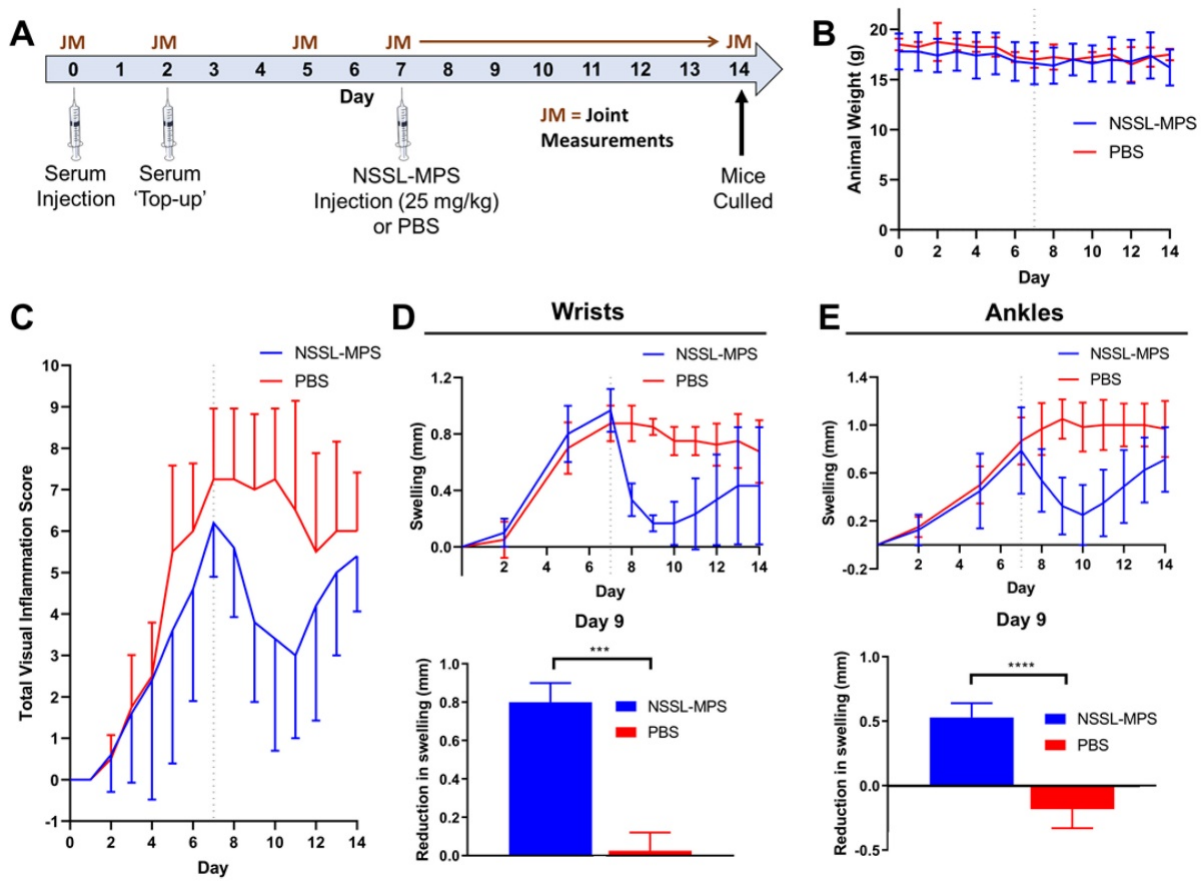
(A) Experimental schedule used for the induction of inflammatory arthritis and subsequent NSSL-MPS therapy study. (B) Animal weights over time for the NSSL-MPS and PBS treated mice; for reference day 7 (day of administration) is shown by the dotted line. (C) Plot of the total visual inflammation scores over time of the NSSL-MPS (n = 5) and PBS (n = 4) treated mice; for reference day 7 is shown by the dotted line. (D) Plot of the wrist swelling of over time for both treament groups (top) and a comparison of the reduction in wrist swelling between days 7 and 9 for both treatment groups (bottom). (E) Plot of the ankle swelling of over time for both treament groups (top) and a comparison of the reduction in ankle swelling between days 7 and 9 for both treament groups. Any joints which did not show signs of inflamation were excluded from the swelling plots. All error bars represent mean ± SD. *** P = 0.0001; **** P = <0.0001.

Having made the correlation between ^89^Zr-NSSL-MPS uptake levels and inflammation in joints in arthritic mice, the therapeutic efficacy of NSSL-MPS was tested in the same STA model. Figure 7A shows the experimental schedule used: STA was induced in healthy female C57Bl/6J mice (n = 9). On day 7 post-serum injection, a therapeutic dose of NSSL-MPS (25 mg/kg) was administered (n = 5), whereas a control group was injected with PBS (n = 4). No adverse effects were seen with either group, based on the lack of weight change (Figure 7B). A clear reduction in the total visual inflammation score was observed for the NSSL-MPS-treated group compared with the PBS group between days 7 - 11 (Figure 7C). The anti-inflammatory effect of NSSL-MPS was more accurately quantified by measuring the joint swelling in the wrists and ankles for both groups (Figure 7D-E; top). In particular, when directly comparing the swelling on day 9 (same timepoint as the PET imaging/biodistribution studies) and day 7 (NSSL-MPS administration), a significantly higher reduction in swelling was observed for the NSSL-MPS treated group (0.8 ± 0.1 mm and 0.5 ± 0.1 mm for wrists and ankles respectively) (Figure 7D-E; bottom). In contrast, swelling either plateaued (0.03 ± 0.10 mm swelling reduction for wrists) or even increased (-0.2 ± 0.1 mm swelling increase for ankles) in the control PBS group (Figure 7D-E; bottom). As expected, the single dose treatment is temporary, as inflammation in all cases resumes with an increase in both inflammation score and joints swelling observed between from day 10 - 11 (3 - 4 days after treatment).

## Discussion

The rationale behind the radiolabelling and *in vivo* tracking of LCLs such as NSSL-MPS is two-fold: *i*) to aid in their pre-clinical development and clinical translation, and *ii*) as potential theranostic agents to support the clinical usage of personalised medicine. Hence, the radiolabelling of a liposomal nanomedicine should ideally not involve any modification of the original structure of the LCL. In addition, it must facilitate the stable incorporation of a radionuclide with a radioactive half-life that matches that of the LCL *in vivo,* due to their long circulating properties. Generally, these properties are accomplished via the use of chelators; either attached to the lipid surface or encapsulated inside the liposomal core. However, even small changes in these modifications, such as the PEG chain length between the chelator and lipid bilayer, can lead to changes in the pharmacokinetics and biodistribution 21. Furthermore, studies comparing surface labelling and the internal trapping of radionuclides using chelators have shown differences in tissue distribution 7,25,26.

^ 89^Zr (*t*_1/2_ = 78.4 h) was chosen among other long-lived positron-emitting radionuclides such as ^64^Cu (*t*_1/2_ = 12.7 h) as it allows *in vivo* tracking over the circulation half-life of LCLs (t_1/2_ of NSSL-MPS = 33 h). In addition, unlike ^64^Cu that shares biodistribution and excretion pathways with liposomes and other nanomedicines (*i.e.* liver), the biodistribution/excretion pathways of ^89^Zr in mice is significantly different. In particular, free ^89^Zr shows mostly bone uptake and no liver accumulation. This facilitates the interpretation of PET imaging studies with nanomedicines and other long-circulating drugs such as antibodies; particularly at late time points when excretion is more likely 7. Thus, we believe these two aspects support the use of ^89^Zr over ^64^Cu for the tracking of liposomes and other nanomedicines *in vivo*. Our radiolabelling method is chelator-free and requires no chemical modification of the liposomal formulation 12. [^89^Zr]Zr(oxinate)_4_ is used to passively transport the radionuclide inside the liposomal core. Once inside, we take advantage of the chelating groups often present on the encapsulated and highly concentrated drug cargo (Figure 1A) to bind to the radiometal and trap it stably. Due to this mechanism, the labelling efficiency and stability using this method is inherently limited by the interaction between the drug and the radiometal. Using IR we show that, in the case of MPS, zirconium binding occurs through the carboxylate present on the hemisuccinate group (Figure S2). As ^89^Zr will likely be in the 4+ oxidation state, coupled with the high concentration of MPS within the liposome core, it is possible that ^89^Zr binds to MPS as a Zr(MPS)_4_ complex. The %LE of NSSL-MPS was found to be 69.3 ± 7.7 % with a serum stability of 87.0 ± 0.4 % after 48 h, which is slightly lower than preceding work using this labelling method. For example, [^89^Zr]Zr(oxinate)_4_ radiolabels PEGylated liposomal alendronate (PLA) with >95 % LE, and >95 % serum stability after 72 h. However, when labelling liposomal doxorubicin (DOXIL) lower %LE (50.6 ± 5.3 %) and stability (80% after 72 h) was observed indicating an expected less favourable radiometal-drug interaction compared to that with alendronate 12. Additionally, we observed a sudden loss of activity from ^89^Zr-NSSL-MPS (*ca.* 15 %) after just 1 h incubation in serum and PBS, followed by minimal losses over the following 48 h. We believe this indicates that a small amount of ^89^Zr is not internalised during labelling and is likely present in the phospholipid bilayer, which allows it to be more easily trans-chelated by serum proteins or DTPA (used in PBS stability). Thus, the %LE of 69.3 ± 7.7 % calculated directly after synthesis and purification, is likely an overestimation, and supports incubation with DTPA at 37 ºC for the purification step. Abou *et al.* previously showed that ^89^Zr could bind directly to the lipid phosphate head groups on liposomes. However, this interaction was shown to be weak, contributing to low stability in both serum and *in vivo* 27. Thus, it is likely that the small amount of weakly bound ^89^Zr found in our studies is associated with the phospholipid phosphate groups. Several other GCs that have been encapsulated in LCLs for the treatment in inflammation and cancer are shown in supplementary Figure S3. Based on our observations using PLA we believe that the phosphate analogues of prednisolone, dexamethasone and budesonide may allow higher radiolabelling yields and stability when radiolabelled with [^89^Zr]Zr(oxinate)_4_. Indeed, inorganic nanoparticles based on a zirconium betamethasone phosphate complex have been previously reported - indicating stability for this Zr-GC interaction 28.

The ability of PET imaging to measure the uptake levels of ^89^Zr-NSSL-MPS in a relevant disease model was evaluated in the K/BxN serum transfer model of human inflammatory arthritis such as RA. This model has been shown to represent several of the hallmarks of RA 23, and was chosen over others (*e.g.* collagen induced arthritis, CIA) for its ability to replicate the effector phase of the disease in several strains of mice, and its rapid and transient effect. The K/BxN serum introduces autoantibodies against glucose-6-phosphate isomerase (GPI) into healthy mice (here C57Bl/6), a commonly found antibody in RA patients. This results in the activation of innate immune cells such as neutrophils, and rapid induction of a transient inflammatory arthritis in fully immunocompetent mice. We were successfully able to track ^89^Zr-NSSL-MPS to inflamed joints as well as the distribution in healthy control mice. Uptake of ^89^Zr-NSSL-MPS was significantly higher in joints with inflammation (3.6 ± 1.5 % ID; 17.4 ± 9.3 % ID/mL) compared to those in control mice where no signs of inflammation were observed (0.5 ± 0.1 % ID, n = 36 & 2.7 ± 1.1 % ID/mL, n = 16). ^89^Zr-NSSL-MPS biodistribution showed common patterns with other LCLs, with major uptake in the organs of the mononuclear phagocytic system, mainly spleen and liver. Additionally, low levels of bone uptake were observed across all groups, primarily in shoulders, knees and spine. We believe this is due to release of small amounts of 'free ^89^Zr' from the LCLs which is known to bind to bone minerals 29, although it is not clear whether this release is occurring whilst circulating in the blood stream, or after uptake in tissues. Since LCLs are well known to be taken up by macrophages 30, we believe the latter is the most likely mechanism. Additionally, uptake of ^89^Zr-NSSL-MPS in the bone marrow, a component of the mononuclear phagocytic system, cannot be ruled out but was not measured. Through both PET image quantification and *ex vivo* biodistribution studies we have shown that there is a clear correlation between increased ^89^Zr-NSSL-MPS uptake and increased swelling in joints, indicating that ^89^Zr-NSSL-MPS uptake also correlates with the extent of inflammation in affected joints.

We have also demonstrated that the biodistribution of 'free ^89^Zr' (as the chloride salt) is distinct from that of ^89^Zr-NSSL-MPS, with high bone uptake (33.0 ± 4.6 % ID/g) and low spleen and liver uptake (3.5 ± 1.3 & 6.0 ± 1.7 % ID/g respectively), which is more than double than with ^89^Zr-NSSL-MPS. It is important to highlight these differences, as increased uptake of 'free ^89^Zr' in the form of [^89^Zr]Zr(oxalate)_4_ in arthritic joints has been previously reported 31. Hence, we also wanted to show that 'free ^89^Zr' released from liposomes did not preferentially accumulate in inflamed tissues. Indeed, we found that there was no correlation when comparing uptake of 'free ^89^Zr' versus joint swelling. Therefore, in this case we believe that the uptake of 'free ^89^Zr' in inflamed joints is likely due to the binding of 'free ^89^Zr' to the bone in joints. Additionally, these results indicate that ^89^Zr-NSSL-MPS uptake is predominantly from the accumulation of the liposomes in inflamed joints and not the result of ^89^Zr being released from liposomes in circulation. Despite this, the potential issue of misinterpretation of images due to the bone uptake of ^89^Zr for imaging liposomes in arthritic joints should not be downplayed.

As shown in Figure 3B, the uptake of ^89^Zr-NSSL-MPS in the knees of two mice (*ca.* 4 % ID, 45 % ID/mL) was over double that of controls (*ca.* 1 % ID, 20 % ID/mL) indicating the potential presence of inflammation. Histology showed the knees were indeed arthritic, highlighting the value of this imaging approach to detect and treat occult sites of inflammation. Whilst the distinction between inflamed and non-inflamed knees was possible using the PET/CT images, in cases where the extent of inflammation in the knee is reduced the elucidation between 'free ^89^Zr' and liposomal ^89^Zr uptake may become more difficult.

After establishing the uptake of NSSL-MPS in arthritic joints using PET imaging, we performed a follow up study to assess the therapeutic efficacy of NSSL-MPS at the same time points of imaging. A clear anti-inflammatory effect was observed from a single dose of NSSL-MPS. This data matches previous work with NSSL-MPS in the adjuvant-induced arthritis (AIA) rat model 15,16. Direct comparisons of the overall therapeutic response should be made with caution however; firstly due to the use of a different animal model and secondly due a difference in treatment regime. Avnir *et al.* administered two doses of NSSL-MPS (10 mg/kg) five days apart and showed a sustained reduction in RA for up to 6 days after administration of the initial dose 15. In contrast, we administered a single 25 mg/kg dose of NSSL-MPS with a peak in reduction of inflammation at 3 - 4 days after which swelling and inflammation gradually began to increase again (Figure 7).

Similarly to this study, Metselaar *et al.* radiolabelled a therapeutic dose (10 mg/kg) of liposomal prednisolone phosphate (LCL-PLP) containing the ^111^In chelator DTPA and imaged in AIA rats 32. Scintigraphic whole body images showed gradual uptake in inflamed paws, peaking at 48 h with *ex vivo* biodistribution at the same point demonstrating higher uptake in the paws of AIA rats compared to healthy controls. Overall paw uptake was <1 % ID/g across all groups, which may seem low compared to the values obtained in this study, but consistent with the larger animals used. Similar to our study (Figure 7D-E), a maximal therapeutic effect was reached at 48 - 72 h post injection of LCL-PLP.

As previously discussed, our radiolabelling method has been successful with a variety of different liposomal nanomedicines. Hence, we believe that our PET tracking method could be used to predict the efficacy of other liposomal GCs (*vide supra*), and in other diseases. In particular, LCL-PLP has previously been investigated clinically for the treatment of atherosclerosis 33. Van der Valk *et al*. confirmed uptake in the atherosclerotic plaques using an anti-PEG antibody which showed co-localisation with macrophages in the lesions. Despite this, patients receiving the LCL treatment showed no reduction in inflammation. The tracking of such LCLs using PET may provide a non-invasive, quantitative way of assessing liposomal uptake in areas of inflammation, for the prediction of treatment. This approach has recently been used in the clinic with cancer patients. In a major milestone in the clinical application of personalised nanomedicine, Lee *et al.* administered HER2-targeted DOXIL (MM-302), radiolabelled with ^64^Cu, into breast cancer patients 10. Image quantification using PET images allowed the classification of patients into two groups based on their lowest lesion uptake. From this a correlation between high ^64^Cu-MM-302 uptake and better treatment response was found.

Finally, there are some limitations in our study worth discussing. First, we believe our PET and therapy studies provide substantial evidence of the effectiveness of the radiolabelling method and preferential accumulation of NSSL-MPS in inflamed joints, resulting in an anti-inflammatory effect. However, we were not able to measure MPS concentration in these joints *ex vivo*. Doing so would provide a more direct link between the PET signal and therapeutic efficacy. Secondly, we were not able to perform combined longitudinal imaging/therapy studies. This would allow us to directly link PET signal with the development of inflammation and ultimately therapeutic efficacy in individual joints, providing a more direct proof that PET imaging with ^89^Zr-NSSL-MPS can be predictive of NSSL-MPS therapy. To address these limitations future studies should include longitudinal PET imaging/therapeutic studies, as well as *ex vivo* analysis of MPS concentration in individual joints. Lastly, there are general concerns about the radiation safety of radiolabelled compounds such as ^89^Zr-NSSL-MPS. However, our previous calculations with similar ^89^Zr-liposomes estimated human radiation doses of 0.9 ± 0.2 mSv/MBq, which is within the range of those reported for ^89^Zr-antibodies in human studies 12. For these reasons, we believe that imaging ^89^Zr-NSSL-MPS has potential for clinical translation, although more detailed imaging studies will have to be carried out in order to accurately calculate the radiation dose in humans.

## Conclusions

We have shown that preformed glucocorticoid-loaded liposomes can be radiolabelled with [^89^Zr]Zr(oxinate)_4_. By using this method, PET imaging was used for the first time to study the biodistribution of a glucocorticoid-loaded liposome, which demonstrated high uptake at visible and occult inflamed sites in an RA model. A follow-up therapy demonstrated a clear reduction in inflammation in mice treated with NSSL-MPS. We believe our liposome labelling technique followed by *in vivo* PET tracking will be a useful tool in the preclinical and clinical development of anti-inflammatory liposomal nanomedicines of this type, and could be used to predict the therapeutic response to NSSL-MPS treatment, as well as with similar nanomedicines, in patients with various inflammatory diseases.

## Supplementary Material

Supplementary figures and tables.

## Abbreviations

CT: computed tomography
DOTA: 1,4,7,10-Tetraazacyclododecane-1,4,7,10-tetraacetic acid
DPSE: distearoylphosphatidylethanolamine
DTPA: diethylenetriaminepentaacetic acid
ELVIS: extravasation through leaky vasculature and subsequent inflammatory cell-mediated sequestration
EPR: enhanced permeation and retention
GC: glucocorticoid
ID: injected dose
i.p.: intraperitoneal
i.v.: intravenous
LCL: long circulating liposomes
MPS: methylprednisolone hemisuccinate
NSSL: nano-sterically-stabilised liposomes
PBS: phosphate buffered saline
PEG: polyethylene glycol
PET: positron emission tomography
PLA: PEGylated liposomal alendronate
RA: rheumatoid arthritis
SPECT: single photon emission computed tomography
