# Supplementary material for: PET Imaging of Liposomal Glucocorticoids using 89Zr-oxine: Theranostic Applications in Inflammatory Arthritis
Source: Theranostics. 2020 Feb 26;10(9):3867–79. doi: 10.7150/thno.40403 (PMC7086351; doi:10.7150/thno.40403)
Supplement: Supplementary file 1 — Supplementary figures and tables. [file thnov10p3867s1.pdf]

Supplementary Information

PET Imaging of Liposomal Glucocorticoids using ^{89}Zr-oxine: Theranostic Applications in Inflammatory Arthritis

P. J. Gawne[1], F. Clarke[2], K. Turjeman[3], A. Cope[2], N. J. Long[4], Y. Barenholz[3#], S. Y. A. Terry[1#] and R. T. M. de Rosales[1#*]

[1] School of Imaging Sciences & Biomedical Engineering, King's College London, St. Thomas' Hospital, London, SE1 7EH, UK.

[2] Centre for Inflammation Biology and Cancer Immunology, King's College London, New Hunt's House, London, SE1 1UL, UK.

[3] Laboratory of Membrane and Liposome Research, Department of Biochemistry, Institute for Medical Research Israel-Canada, Hebrew University-Hadassah Medical School, Jerusalem, Israel.

[4] Department of Chemistry, Imperial College London, White City Campus, W12 0BZ, London, UK

* Corresponding author: rafael.torres@kcl.ac.uk

[#] These authors contributed equally

Materials and methods

All chemical reagents were purchased from commercial sources. Water (18.2 MΩ·cm) was obtained from an ELGA Purelab Option-Qsystem. UV titrations were carried out using a PerkinElmer Lambda 25 spectrometer, with samples in Brand 70 µL micro cuvettes. No-carrier-added 89Zr (produced at the BV Cyclotron, VU Amsterdam, NL) was purchased from PerkinElmer as $[^{89}Zr]Zr(oxalate)_4$ in 1 M oxalic acid. Radioactivity in samples were measured using CRC-25R dose calibrator (Capintec). iTLC-SG and SA strips were purchased from Agilent, UK and scanned using the PerkinElmer Cyclone Plus Storage Phosphor Imager. Gamma counting was performed using a Wallac 1282 CompuGamma γ counter. The human biological samples were sourced ethically and their research use was in accord with the terms of the informed consents under an Institutional Review Board/Ethics Committee (IRB/EC) approved protocol. All animal studies were ethically reviewed and carried out in accordance with the Animals (Scientific Procedures) Act 1986 and the GSK Policy on the Care, Welfare and Treatment of Animals.

$[^{89}Zr]Zr(oxinate)_4$ radiosynthesis

$[^{89}Zr]Zr(oxalate)_4$ (5−75 MBq) was transferred to a 1.5 mL plastic vial, diluted to 100 µL with water (18.2 MΩ·cm) and gradually adjusted to pH 7.5−8 with 1 M sodium carbonate. The volume was then adjusted to 450 µL with Chelex®-treated water and 50 µL of a 10 mg/mL solution of 8-hydroxyquinoline in chloroform was added. The mixture was vortexed for 5 min, a further 450 µL of chloroform were added and the mixture vortexed for 10 min. The organic phase was extracted into a conical glass vial and dried at 60 °C under a flow of nitrogen gas. The residue was dissolved in aqueous dimethyl sulfoxide (DMSO) for further use. Radiochemical yield was defined by the amount of radioactivity present in the dried organic extract divided by the starting amount of radioactivity. Radioactivity in samples was measured with a CRC-25R dose calibrator (Capintec).

Characterisation of the Zr-MPS complex

10 mg/mL solutions of methylprednisolone sodium hemisuccinate (MPS-Na) and non-radioactive $ZrCl_4$ in MeOH were prepared. A 6 µL aliquot of $ZrCl_4$ (60 µg, 0.26 µmol, 0.5 equivs.) was added to a 25 µL solution of MPS-Na (0.2 mg, 0.5 µmol, 2 equivs.) and the solution heated at 50 ºC for 30 mins. The solution was applied to the ATR diamond on an ATR-IR system and the solvent removed via mild heating with a heat gun. This process was repeated until a thin film was visible on the ATR diamond. The IR spectrum of the mixture was then taken. For an IR of MPS-Na, aliquots of the 10 mg/mL solution of MPS-Na was applied to the ATR diamond instead.

Dynamic light scattering (DLS) measurements of NSSL-MPS before and after radiolabelling

For size and polydispersity measurements:- After radiolabelling an aliquot of ^{89}Zr-NSSL-MPS was diluted 10x in 10% PBS in water and the size and polydispersity measured by DLS. For control measurements, an aliquot of NSSL-MPS was diluted in saline to match the same concentration of ^{89}Zr-NSSL-MPS after radiolabelling. An aliquot of this was then diluted 10x in 10% PBS in water and the size and polydispersity measured by DLS.

For zeta-potential measurements:- The same solutions used for the above size and polydispersity measurements were diluted a further 5x in 10% PBS in water and the zeta potential measured by DLS.

[^{89}Zr]$ZrCl_4$ ('free ^{89}Zr') radiosynthesis

[^{89}Zr]Zr(oxalate)$_4$ (55 MBq) was transferred to a 1.5 mL plastic vial, diluted to 300 µL with water (18.2 MΩ·cm). The solution was then loaded onto a QMA cartridge (Sep-Pak Light Plus, Waters) - which had been washed with ethanol (5 mL), saline (10 mL) then water (10 mL,18.2 MΩ·cm) – and the column washed with water (1 mL,18.2 MΩ·cm) and then 1 M HCl (150 µL). [^{89}Zr]$ZrCl_4$ (54 MBq) was obtained by elution with a further 1 M HCl wash (350 µL). [^{89}Zr]$ZrCl_4$ (7.5 MBq, 50 µL) was then diluted with saline (350 µL) and then neutralised (measured with pH strips) by addition of 1 M sodium carbonate (24 µL). Saline (76 µL) was added and the solution passed through a 0.2 µm filter – ready for injection.

Biodistribution of free ^{89}Zr in RA mice

Biodistribution studies were carried out in accordance with British Home Office regulations governing animal experimentation. Rheumatoid arthritis was induced in female 9-week old C57Bl/6 mice (n = 2) as described in the main text, with visual inflammation scores assigned on each day post-serum injection and caliper measurements being performed on the wrists and ankles on Days 0, 2, 5, 7 & 9. At day 7 post-serum injection, all mice were anesthetised with isofluorane (1.5−2%) and [^{89}Zr]$ZrCl_4$ (1.6-1.8 MBq, 120 µL) was injected *i.v.* into the mice. Mice were culled by cervical dislocation at 48 h p.i. whilst under anaesthesia, and the organs of interest were dissected, weighted and gamma-counted together with standard samples of the injected radiotracer to obtain percentages of the injected dose per mass values (%ID/g) for each organ/tissue (Figure 6A). Each sample was weighed and counted with a γ counter (LKB compugamma), together with standards prepared from a sample of the injected [^{89}Zr]$ZrCl_4$.

Table 1: Raw values for the hydrodynamic diameter (size, nm), zeta-potential (surface charge, mV) and polydispersity index of NSSL-MPS before and after radiolabelling.

	**Hydrodynamic diameter (nm)**		**Zeta-potential (mV)**		**Polydispersity (PDI)**	
	Before labelling	After labelling	Before labelling	After labelling	Before labelling	After labelling
	78.1	80.2	-4.8	-2.1	0.085	0.101
	77.4	78.6	-6.0	-2.9	0.081	0.100
	75.4	77.7	-6.9	-5.4	0.072	0.084
**Mean ± SD**	**77.0 ± 1.4**	**78.8 ± 1.2**	**-5.9 ± 1.1**	**-3.5 ± 1.8**	**0.079 ± 0.007**	**0.095 ± 0.01**

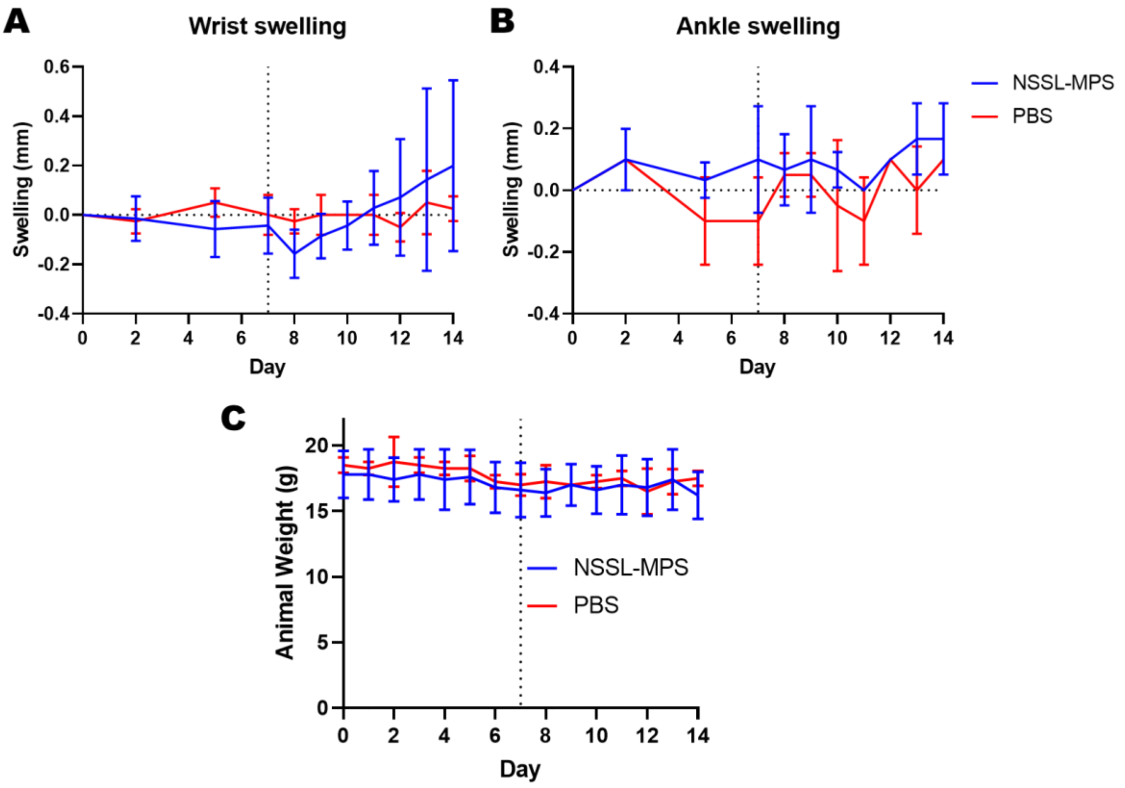

Figure S 1: Plots of joint swelling over time for ankles (A) and wrists (B) for joints excluded from the plots in Figure 7 in the main text. C) weights of mice given NSSL-MPS (25 mg/kg) and PBS over time.

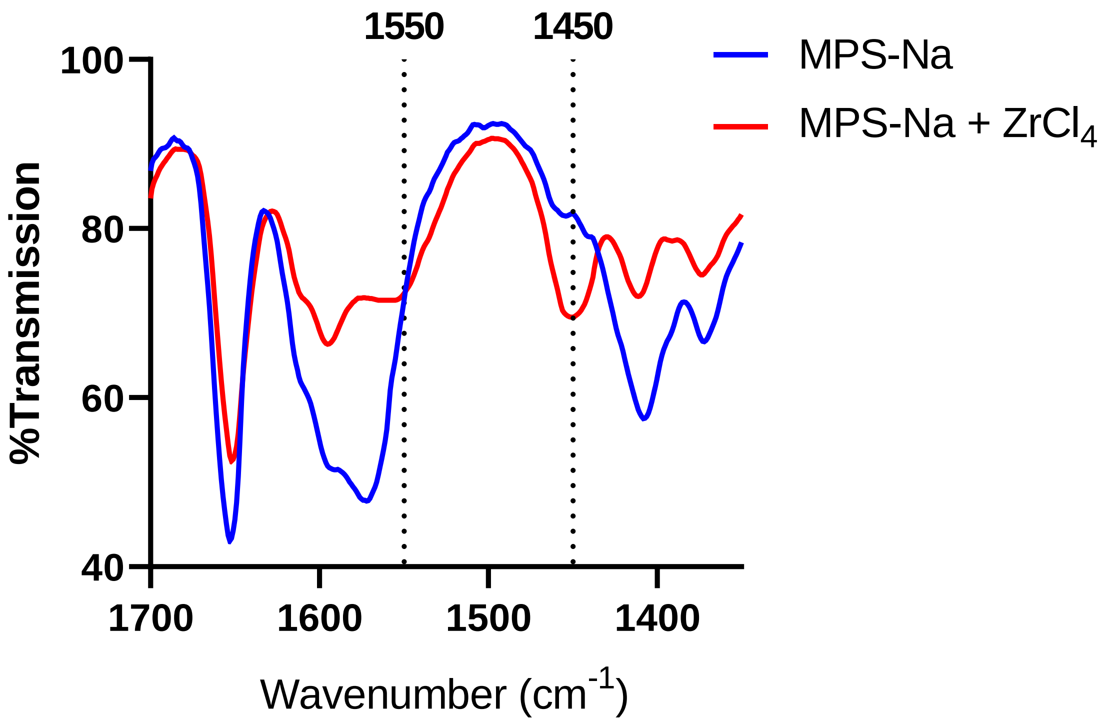

Figure S 2: Infra-red spectrum of MPS-NA (blue line) compared with that of a solution containing MPS-Na and ZrCl4 (red line). New peaks formed at 1450 cm-1 ¬and 1550 cm-1 corresponding to COO-Zr carboxylate salt.

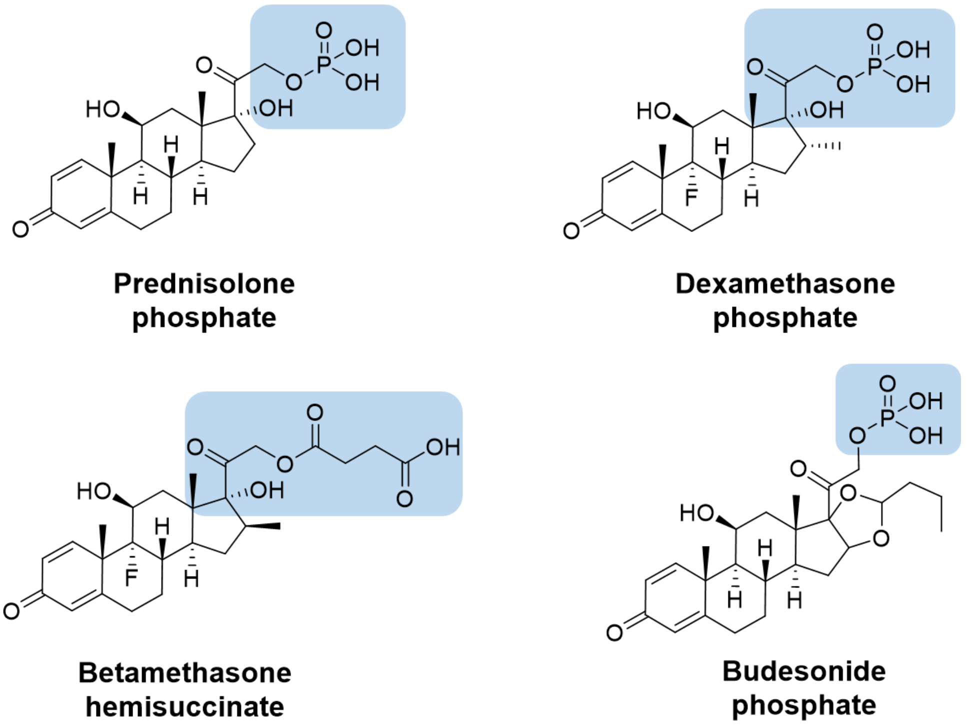

Figure S 3: Chemical structures of other glucocorticoids which have liposomal formulations.

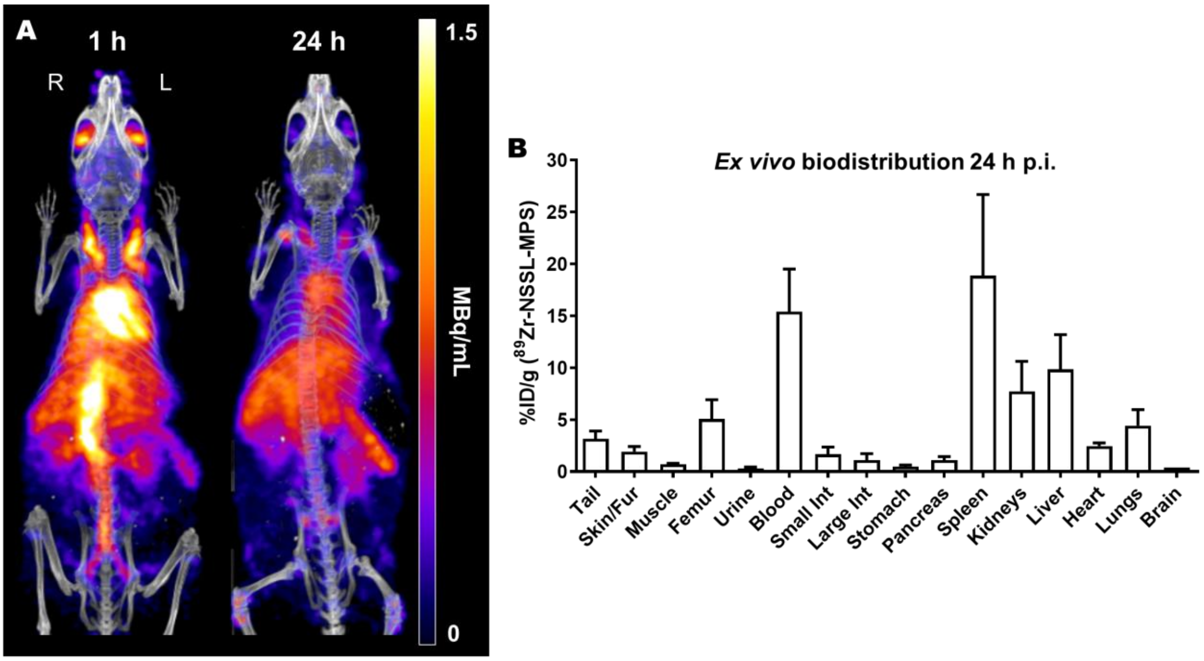

Figure S 4: A) PET/CT maximum intensity projections of CD1 female mice injected with ^{89}Zr-NSSL-MPS (1 MBq) at 1 h post injection (p.i.; left image) and 24 h p.i. (right image). B) *Ex vivo* biodistribution of ^{89}Zr-NSSL-MPS 24 h p.i.
